# Probing corrosion using a simple and versatile in situ multimodal corrosion measurement system

**DOI:** 10.1038/s41598-023-42249-0

**Published:** 2023-10-04

**Authors:** Sridhar Niverty, Rajib Kalsar, Robert J. Seffens, Anthony D. Guzman, Timothy J. Roosendaal, Lyndi Strange, Vineet V. Joshi

**Affiliations:** Energy and Environment Directorate, Pacific Northwest National Laboratory, Richland, WA 99354 USA

**Keywords:** Metals and alloys, Characterization and analytical techniques

## Abstract

In this work, we have developed a unique in situ multimodal corrosion system that is capable of acquiring electrochemical data, sample imaging/visualization and hydrogen collection, simultaneously. Each of these modalities yield valuable information pertaining to the ongoing corrosion process. Combining them can yield holistic information on the role of microstructure, processing history, presence of coatings, etc., on the sequence of steps occurring during the corrosion process, and how they correlate with the acquired electrochemical data. Four materials systems, namely AA6061-T6 aluminum alloy, AZ91 magnesium alloy, galvanized DP590 steel, and pure Zn, were investigated under open circuit potential and under potentiodynamic polarization. The multimodal corrosion system was utilized to observe processes such as surface passivation and dissolution, pit and filiform corrosion initiation and propagation, and was correlated with location and magnitude of hydrogen evolution. This approach is shown to yield a truly multimodal understanding of the ongoing corrosion processes.

## Introduction

Corrosion analysis is fundamental to determining and predicting the performance of material systems. The corrosion behavior of materials is determined by their composition, processing history, microstructure and service environment. Intermetallic particles such as inclusions (metallic and non-metallic), precipitates, and dispersoids can be present and exhibit unique electrochemical behaviors depending on their chemical composition and the corrosive environment^1–5^. Furthermore, microstructural characteristics such as grain orientation, grain texture, surface roughness, presence of distinct phases, and sample/part geometry contribute to corrosion susceptibility^6,7^. Finally, the presence of dissimilar material combinations can further exacerbate the corrosion behavior of the electrochemically anodic material^8,9^.

To quantify corrosion damage, several measurement methods have been developed. They broadly involve using samples of the material or component to be tested, electrochemical measurement tools (e.g. polarization curves) to quantify ongoing corrosion behavior, corrosion by-product collection methods (e.g. evolved hydrogen collection), imaging methods (2D or 3D), and cells/chambers (e.g. salt fog testing) designed to subject a material to a fixed environment or a combination of predetermined environments (e.g. dry–wet cycling)^10–16^. Electrochemical characterization usually involves the use of a working electrode (sample to be tested), a reference electrode, counter electrode, and a power source (i.e. potentiostat). It encompasses several destructive and nondestructive capabilities that can test materials under open circuit and accelerated conditions. These capabilities make potentiostat assisted electrochemical characterization ideal for studying corrosion phenomena such as oxide film breakdown, metastable, and stable pitting^7,17^. However, electrochemical methods are often used independently of other techniques to obtain information pertaining to ongoing corrosion processes. For example, Potentiodynamic Polarization (PD) measurements often involve large changes to the net corrosion current (over several orders of magnitude) over a dynamically applied potential. The effect of large changes in the net current on the microstructure can be challenging to interpret without correlative characterization techniques such as time resolved 2D or 3D imaging. This can lead to difficulty with identification of initiation site(s), role of phases as cathodes or anodes, susceptibility of interfaces such as grain boundaries, role of gradient microstructures and the sequence of events occurring during the corrosion process. In turn, this can lead to an oversimplified interpretation of the ongoing corrosion process. Materials containing significant microstructural heterogeneity (examples: friction stir processed materials, functionally graded materials, multiphase materials or multimaterial joints) can be particularly challenging to study due to the absence of corroborating information that describe the role of specific phases or interfaces^18,19^. An approach to overcome this challenge involves investigating the role of microstructural constituents using a combination of pre- and post-corrosion imaging methods or interrupting the ongoing corrosion process. However, these methods yield information about the corrosion process before and after the corrosion process, leaving behind vital information about microstructural changes taking place *during* the corrosion process. Thus, characterization that informs of the sequence of microstructural events taking place *during* the corrosion process can be invaluable to gain a holistic view of how corrosion initiates and progresses.

**Figure 1:**
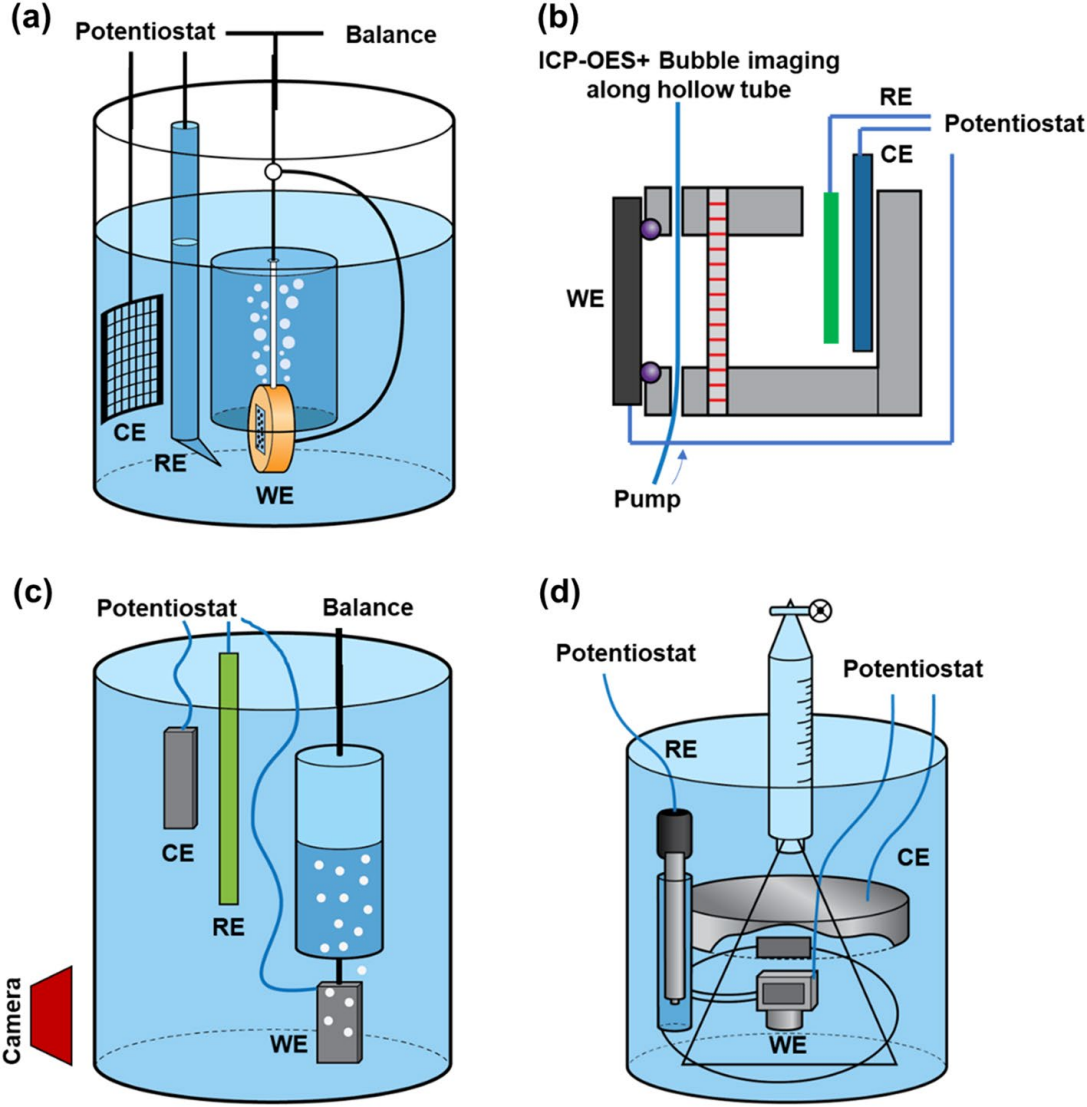
Reported examples of different corrosion setups aimed at acquiring data in addition to electrochemical data during the corrosion of materials. (**a**) Fajardo et al. used a gravimetric method for calculating amount of hydrogen collected^20–22^, (**b**) Lebouil et al. used atomic emission spectro-electrochemistry and imaged bubbles emitted from the surface^23^, (**c**) Curioni et al. used sample imaging and hydrogen collection^24,25^, (**d**) Frankel et al. used evolved hydrogen volume measurement^26^.

In this study, a simple and portable corrosion apparatus has been developed and utilized to obtain corrosion data from multiple complementary modalities simultaneously, namely, electrochemical testing and in situ visualization of multiple materials of interest (in 2-D) and to capture evolved hydrogen. Examples of previously developed setups to acquire supplementary corrosion information along with electrochemical data are shown in Fig. 1^20–26^. The multimodal corrosion system introduced in this study is an evolution in design inspired from the advantages and disadvantages of these previously developed setups. It is important to note that other designs for monitoring corrosion in situ exist, which can be used to perform a variety of analyses such as, investigate crevice corrosion using visual observation, or utilize Raman Spectroscopy for solid–liquid reactions analysis^27,28^. While each of these systems have significant merit, the aim of the system developed in this manuscript was to enable hydrogen evolution, visual observation and electrochemical measurements in a rapid and reproducible manner. The system can be used to analyze corrosion in epoxy mounted or unmounted samples and is agnostic to sample size and geometry/shape. It can be used to capture high resolution time lapse images of the corroding surface through a viewing window. The system also eliminates the need for suspending cables within hollow glass rods and insulating paint or tape around the full surface of the sample mount. Instead, they are replaced by a simple threaded aluminum rod that is in constant contact with the sample and passes through the mount and back wall of the bath. This also ensures fewer components inside the bath, thereby easing startup and reproducibility of corrosion experiments. The compact sample mounting setup also allows for rapid change of samples after each corrosion test and ensures that the sample surface always faces the camera.

The system is also being further developed for evolved gas composition analysis, corrosion area analysis, and to track solution pH and conductivity, simultaneously, thus further adding to its versatility and capabilities. In this study, Open Circuit Potential (OCP) and PD scans were performed in sequence with particular interest in delineating the microstructural evolution taking place during the different stages of the PD curve in real time. Examples from four materials systems (galvanized dual phase 590 steel, magnesium alloy AZ91, aluminum alloy AA6061, and commercially pure zinc) are demonstrated and their implications discussed. The aim of this manuscript is to discuss the many benefits that can be reaped from the combination of surface visualization, hydrogen collection and electrochemical measurement. Aspects pertaining to correlating the microstructure and processing history with the observed corrosion attack will be addressed in future manuscripts dedicated to these (and other) materials individually and their respective processing histories.

## Methods

**Figure 2:**
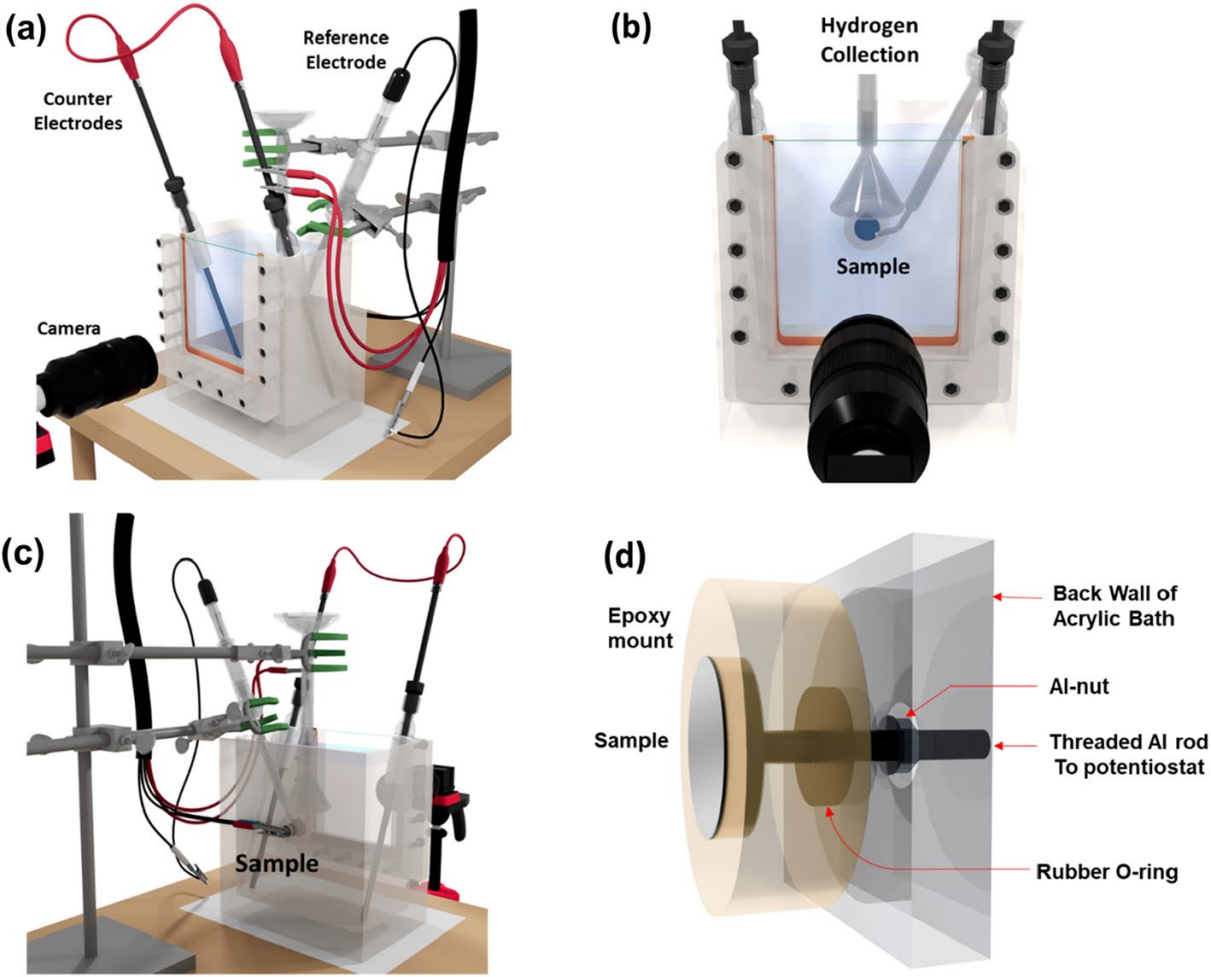
(**a**–**c**) Schematics showing the multimodal corrosion system from different viewing angles. (**d**) Schematic showing a side view of the mounted sample inside the chamber and the fastened threaded rod connecting the sample to the outside of the acrylic bath to be connected to a potentiostat.

Figure 2a–c show schematic diagrams of the system from different directions. Furthermore, a schematic diagram showing the top and side views, and dimensions of the bath is shown in the supplementary section (Supplementary Fig. S1). The system consists of an acrylic corrosion bath with a viewing window. The sample to be corrosion tested is mounted or glued to an epoxy mount with the region of interest facing the camera (on the opposite face of the viewing window). The sample can also be used as-received or after polishing polishing to a desired surface finish. The opposite end of the sample facing the epoxy mount is connected to a threaded aluminum rod drilled into the epoxy from the bottom (Fig. 2d). In the case where a sample is glued to the epoxy mount, superglue is used to bond the sample with the mount and lacquer is used to protect the sides or edges of the sample. This controls the area exposed to the fluid and prevents solution ingress inside and under the mount. A rubber O-ring enables the back of the epoxy mount to form a hermetic seal with the acrylic wall. The aluminum rod is passed through the O-ring and through a hole in the acrylic wall. A nut on the outside of the bath is fastened around the threaded rod to compress the O-ring and thereby prevents leakage of the corrosive fluid. The threaded rod sticking out of the back of the acrylic bath is connected to a potentiostat (Fig. 2d). A high-resolution camera is used to visualize the corrosion in the region of interest from the front, i.e. through the viewing window. Hydrogen bubbles evolved during the corrosion are captured using a funnel and measuring cylinder. Details of each of the components of the apparatus are given below:

1. Potentiostat—A Gamry G750 potentiostat was used for these studies. For all the samples studied, an OCP measurement was performed for 1800 s, following which PD measurement was performed. A saturated calomel electrode was used as the reference electrode and graphite rods were used as counter electrodes. The reference electrode was placed at a distance of less than 1 mm from the sample. A scan rate of 10 mV/min was used for all the potentiodynamic polarization experiments.
2. Imaging system—A Tokina atx-i macrolens was utilized to perform high resolution imaging of the sample surface during the corrosion process. Images were obtained at regular intervals of 5 s during the entire corrosion duration, compiled together into an image stack and correlated with the electrochemical data. ImageJ was utilized to crop the images acquired during corrosion, insert scale bars and to compile the imaging data into videos^29^.
3. Hydrogen collection—A funnel and inverted measuring cylinder were used to capture and measure evolved hydrogen gas.

## Results

### Case I: Aluminum alloy (AA6061-T6)

Hot rolled AA6061-T6 was obtained as 3 mm thick sheets and punched into circular samples with a diameter of 15.875 mm. These circular samples were epoxy mounted, and their surface was polished to a 1 µm diamond finish. OCP measurement was performed for a duration of 1800 s and was followed by PD measurement from 200 mV below OCP up to 200 mV above OCP in 3.5 wt% NaCl solution.

**Figure 3:**
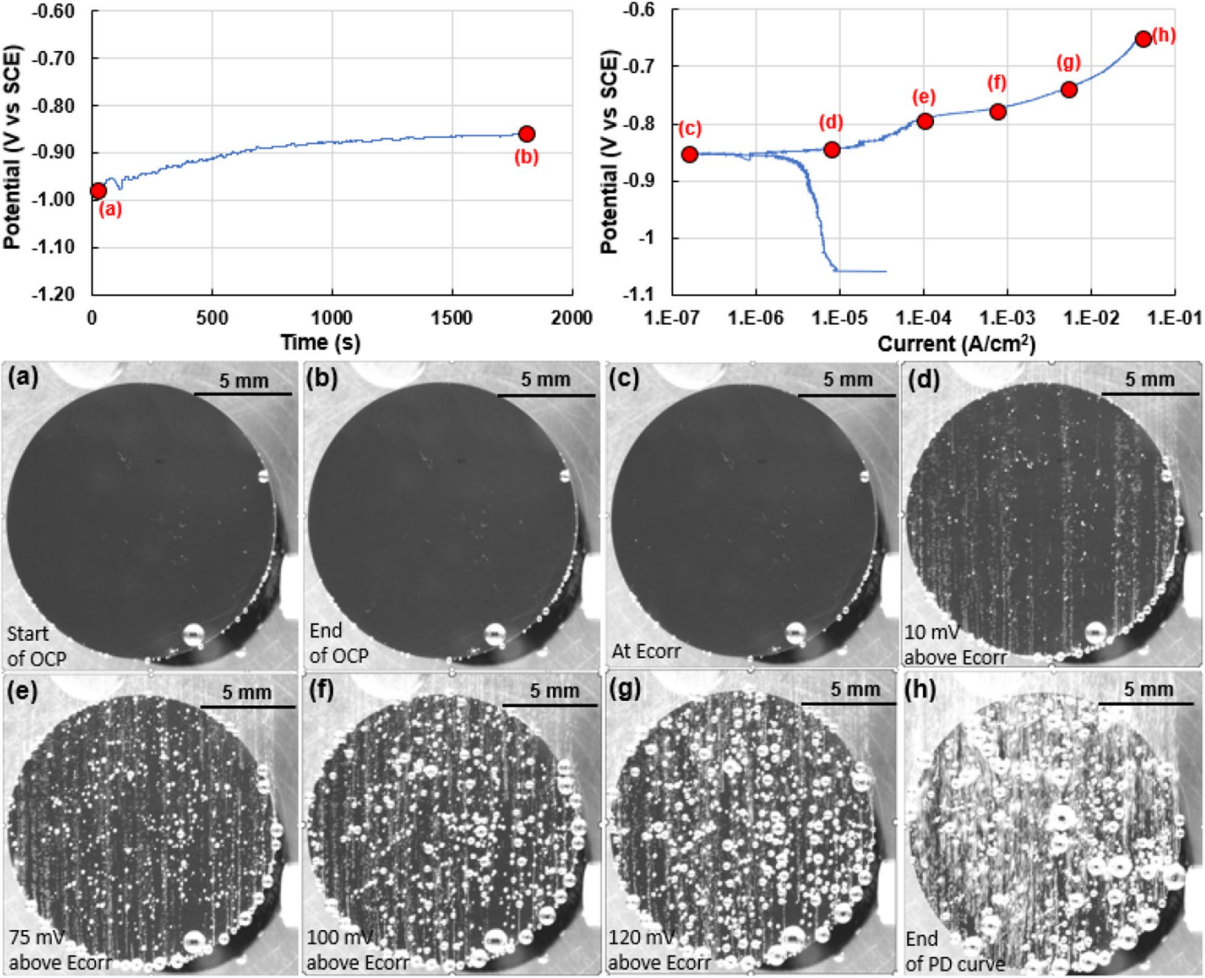
Representative OCP (potential (V vs SCE) vs time(s)) and PD curves (potential (V vs SCE) vs current (A/cm^2^)) of AA6061 aluminum alloy. Annotations in red highlight locations where images of the sample surface are shown, i.e. (**a**) at the beginning of OCP, (**b**) end of OCP, (**c**) at E_corr_, (**d**) at an applied potential of 10 mV above E_corr_ with current density of 1E-04 A/cm^2^, (**e**) at an applied potential of 75 mV above E_corr_, (**f**) at an applied potential of 100 mV above E_corr_, (**g**) at an applied potential of 120 mV above E_corr_, (**h**) at the end of the recorded PD curves showing rapid hydrogen evolution. Images show the increase in corrosion as well as hydrogen bubble evolution as a function of imposed potential.

Figure 3 shows representative OCP and PD curves for AA6061. Annotations (red color lettering from (a) to (h)) on these plots indicate locations from where images of the corroding sample are presented. Aluminum AA6061 undergoes no changes to the surface during the OCP measurement (inset Fig. 3a and b).

A marginal amount of hydrogen release was observed during the cathodic polarization curve, as shown in image 3(c). Hydrogen bubble release was observed to increase drastically after the onset of the anodic polarization curve (Fig. 3d–h). The number of sites and volume of hydrogen released increased with increase in potential. Sites of hydrogen bubble nucleation and release were associated with the location of pits. The number density and size of pits increased with increase in potential and led to a greater volume in hydrogen bubble evolution. Corroding over this range of potentials, a total of 2 ml of hydrogen was captured during the PD measurement. The possible set of corrosion reactions are shown below. Included is the oxygen reduction reaction owing to the presence of dissolved oxygen in the electrolyte. It is not possible to determine the dominant cathodic reaction from using the multimodal system in its current configuration. A video correlating the OCP and PD curves with the video of the corroding sample is included in the supplementary section (AA6061_MmC corrosion.mp4).

$${\text{Anodic reaction}}:\,\,\,{\text{2Al}} \to 2{\text{Al}}^{{{3} + }} + {\text{6e}}^{ - } ,$$

$$\begin{aligned} {\text{Cathodic reactions}}: \, & {\text{HER}}:{\text{ 2H}}_{{2}} {\text{O }} + {\text{ 2e}}^{ - } \to {\text{2OH}}^{ - } + {\text{ H}}_{{2}} \uparrow \\& {\text{ORR}}:{\text{ O}}_{{2}} + {\text{ 4e}}^{ - } + {\text{ 2H}}_{{2}} {\text{O}} \to 4{\text{OH}}^{ - } , \\ \end{aligned}$$

$${\text{Net redox reaction}}:{\text{ 2Al }} + {\text{ O}}_{{2}} + {\text{ 6 H}}_{{2}} {\text{O}} \to {\text{2Al}}\left( {{\text{OH}}} \right)_{{3}} + {\text{ 3H}}_{{2}} \uparrow .$$

### Case II: Magnesium alloy (AZ91)

**Figure 4:**
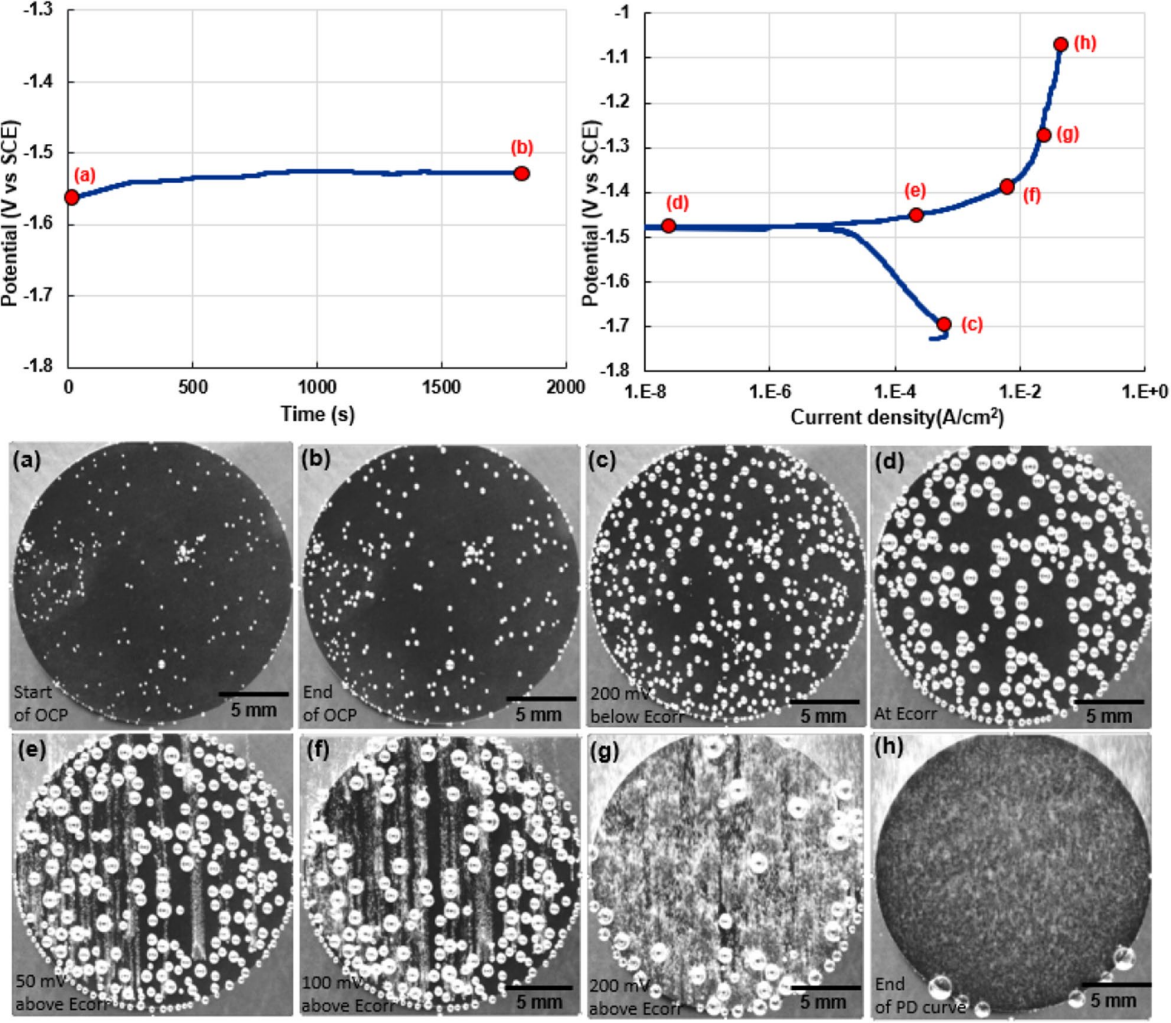
Representative OCP (Potential (V vs SCE) vs time(s)) and PD curves (potential (V vs SCE) vs current(A/cm^2^)) of AZ91 alloy. Annotations highlight locations where images of the sample surface are shown, i.e. at (**a**) the beginning of OCP, (**b**) the end of OCP, (**c**) the start of the cathodic curve, (**d**) E_corr_, (**e**) a potential of ~ 50 mV above E_corr_, (**f**) a potential of ~ 100 mV above E_corr_, (**g**) a potential of ~ 200 mV above E_corr_, (**h**) end of PD curve. Images show the increase in corrosion as well as hydrogen bubble evolution as a function of imposed potential.

High pressure die cast (HPDC) AZ91 was procured in the form of 3 mm thick sheets and punched into circular samples with a diameter of 15.875 mm. These samples were epoxy mounted with their surface polished to a 1 µm diamond finish. OCP measurement was acquired for a duration of 1800 s following which PD measurement was performed from 200 mV below OCP up to 500 mV above OCP. Figure 4a and b show the OCP and PD measurement curves obtained for the AZ91 in 0.6 wt% NaCl solution. Figure 4a and b also show locations on the OCP and PD curves where snippets of the sample video are included. These locations highlighted show the sample at (a) the beginning of OCP, (b) the end of OCP, (c) 200 mV below E_corr_, (d) E_corr_, (e) 50 mV over E_corr_, (f) 100 mV over E_corr_, (g) 200 mV over E_corr_ and, (e) a potential of − 1.05 V or the potential at which the PD test was terminated. These locations were chosen due to the drastic difference in the microstructural features observed at these stages of corrosion. Hydrogen evolution in the form of bubbles is an excellent indicator of the presence of and rate of corrosion reaction taking place in magnesium alloys.

Inset Fig. 4a and b show the corrosion attack of AZ91 during 1800 s under OCP. During OCP, the magnitude of corrosion taking place is very minimal, as indicated by the small increase in size of corrosion bubbles on the surface of the sample. Comparing inset Fig. 4b–d shows an increase in size and number of bubbles seen on the surface during the cathodic polarization curve. The number of corrosion sites and magnitude of evolved hydrogen increased drastically with applied potential (inset Fig. 4e and f). The locations of the sample with a large magnitude of surface corrosion (such as pits) were also the locations that show a greater magnitude of hydrogen bubble release. Larger bubbles evolved from locations where the surface corrosion rate was slow. With increase in potential, the entire sample surface was observed to undergo rapid corrosion as shown in inset Fig. 4g and h. This was indicated by the release of small but fast evolving hydrogen bubbles that encompassed the entire surface of the sample. A video correlating the OCP and PD curves with the video of the corroding sample is included in the supplementary section (AZ91_MmC corrosion.mp4).

The possible set of corrosion reactions are shown below. Included is the oxygen reduction reaction owing to the presence of dissolved oxygen in the electrolyte. It is not possible to determine the dominant cathodic reaction from using the multimodal system in its current configuration^26^:

$${\text{Anodic reaction}}:\,\,\,\,{\text{Mg}} \to {\text{Mg}}^{{{2} + }} + {\text{ 2e}}^{{^{ - } }} ,$$

$$\begin{aligned} {\text{Cathodic reactions}}:\,\,\,\,\, & {\text{HER}}:{\text{ 2H}}_{{2}} {\text{O }} + {\text{ 2e}}^{ - } \to {\text{2OH}}^{ - } + {\text{ H}}_{{2}} \uparrow \\& {\text{ORR}}:{\text{ O}}_{{2}} + {\text{ 4e}}^{ - } + {\text{ 2H}}_{{2}} {\text{O}} \to {\text{4 OH}}^{ - } , \\ \end{aligned}$$

$${\text{Net redox reaction}}:{\text{ 3Mg }} + {\text{ O}}_{{2}} + {\text{ 4H}}_{{2}} {\text{O}} \to {\text{3Mg }}\left( {{\text{OH}}} \right)_{{2}} + {\text{ H}}_{{2}} \uparrow .$$

**Figure 5:**
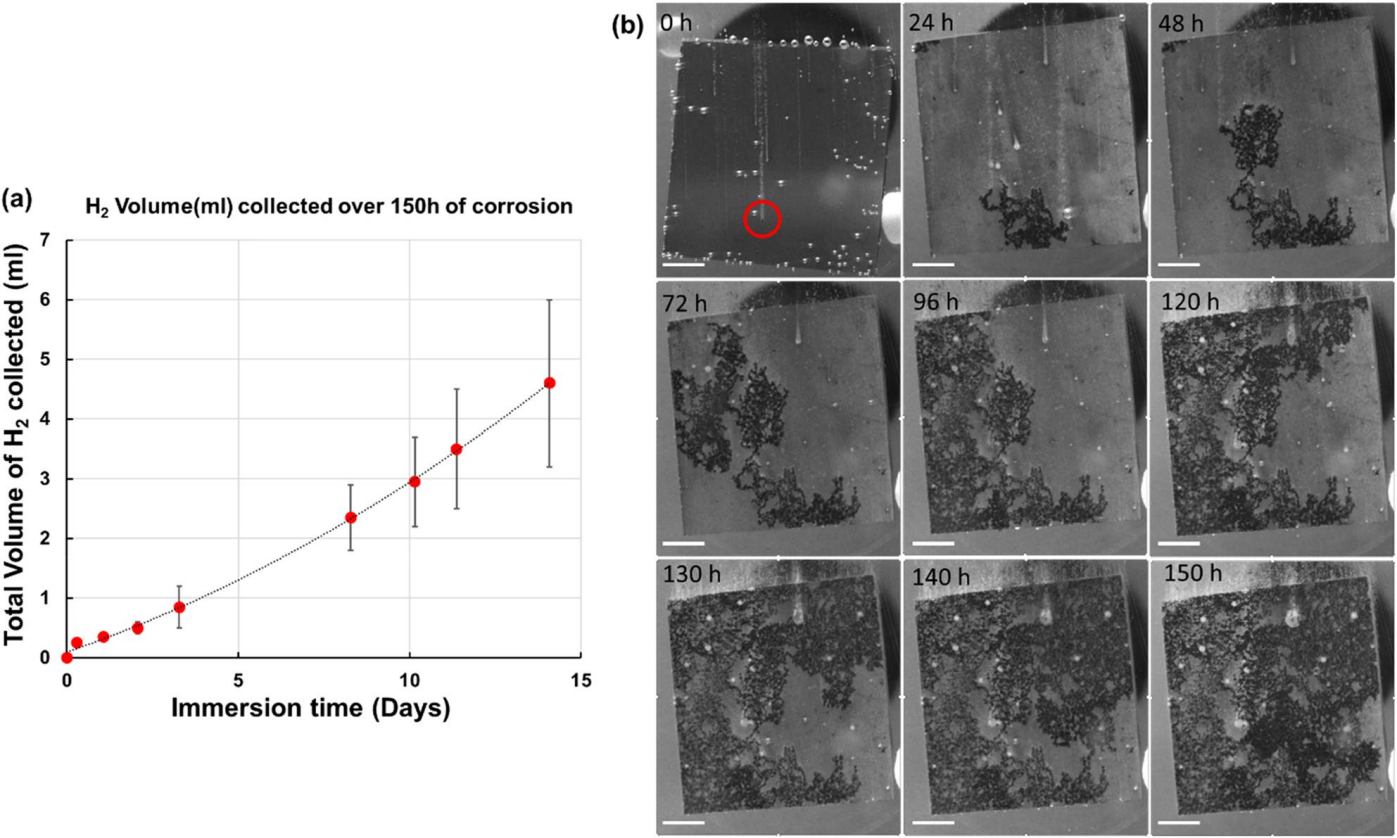
(**a**) Evolved hydrogen gas as a function of immersion corrosion duration over 2 weeks of immersion in 0.6 wt% NaCl solution, (**b**) images of the corroding AZ91 sample as a function of immersion time showing the initiation (red circle highlighting a pit that formed immediately upon immersion) and progress of corrosion damage accompanied by the release of hydrogen bubbles. The corrosion front is the largest source of hydrogen gas, followed by the dark corroded regions. Scale bars correspond to 2 mm in length.

In both the AA6061 and AZ91 cases, the size, number, and rate of hydrogen bubble release from specific sites of the microstructure are visual indicators for qualitatively deducing the rate of ongoing corrosion. Figure 5 shows an example of corrosion in AZ91 magnesium alloy under OCP in 0.6 wt% NaCl solution over a duration of 338 h (2 weeks). During this process, filiform corrosion and pitting were the dominant forms of corrosion. Figure 5a shows a plot of total hydrogen released from the sample as a function of corrosion time. The snippet images from the ongoing corrosion depict corrosion attack of the surface as a function of immersion time (Fig. 5b). Immediately upon immersion, corrosion was initiated on the surface by the formation of a pit and accompanying burst of rapid (small in bubble volume and large number of bubbles) hydrogen release (red circle annotation in 0 h image of Fig. 5b). Following initiation, the entire surface of the sample progressively underwent filiform corrosion over 150 h. The rate of hydrogen bubble evolution seen at the filiform corrosion fronts was greater than the rate of hydrogen evolution seen in pristine areas of the sample or in the previously corroded areas (i.e. regions of the sample image characterized by dark gray values). This demonstrates visually that a greater magnitude of localized corrosion attack is accompanied by a greater volume and higher rate of small hydrogen bubbles being formed and released. Additionally, areas with lower magnitude of corrosion showed a lower hydrogen evolution rate, larger hydrogen bubbles, and minimal nearby matrix dissolution. A video correlating the OCP and PD curves with the video of the corroding sample is included in the supplementary section (AZ91-MmC corrosion-150 h OCP.mp4).

### Case III: Galvanized dual phase steel (DP590)

A circular sample (diameter of 15.875 mm) of galvanized dual phase (DP590) was glued to an epoxy sample mount using super glue. Lacquer was used to coat the edges of the sample to prevent the ingress of the corrosive solution underneath the galvanized DP590 sample. The surface of the sample was left unprepared, i.e. the sample was not ground or polished to avoid the removal of the galvanized Zn layer. OCP measurement was performed for a duration of 1800 s, after which PD measurement was performed from a potential of 200 mV below the OCP and up to 400 mV over the OCP in 3.5 wt% NaCl solution.

**Figure 6:**
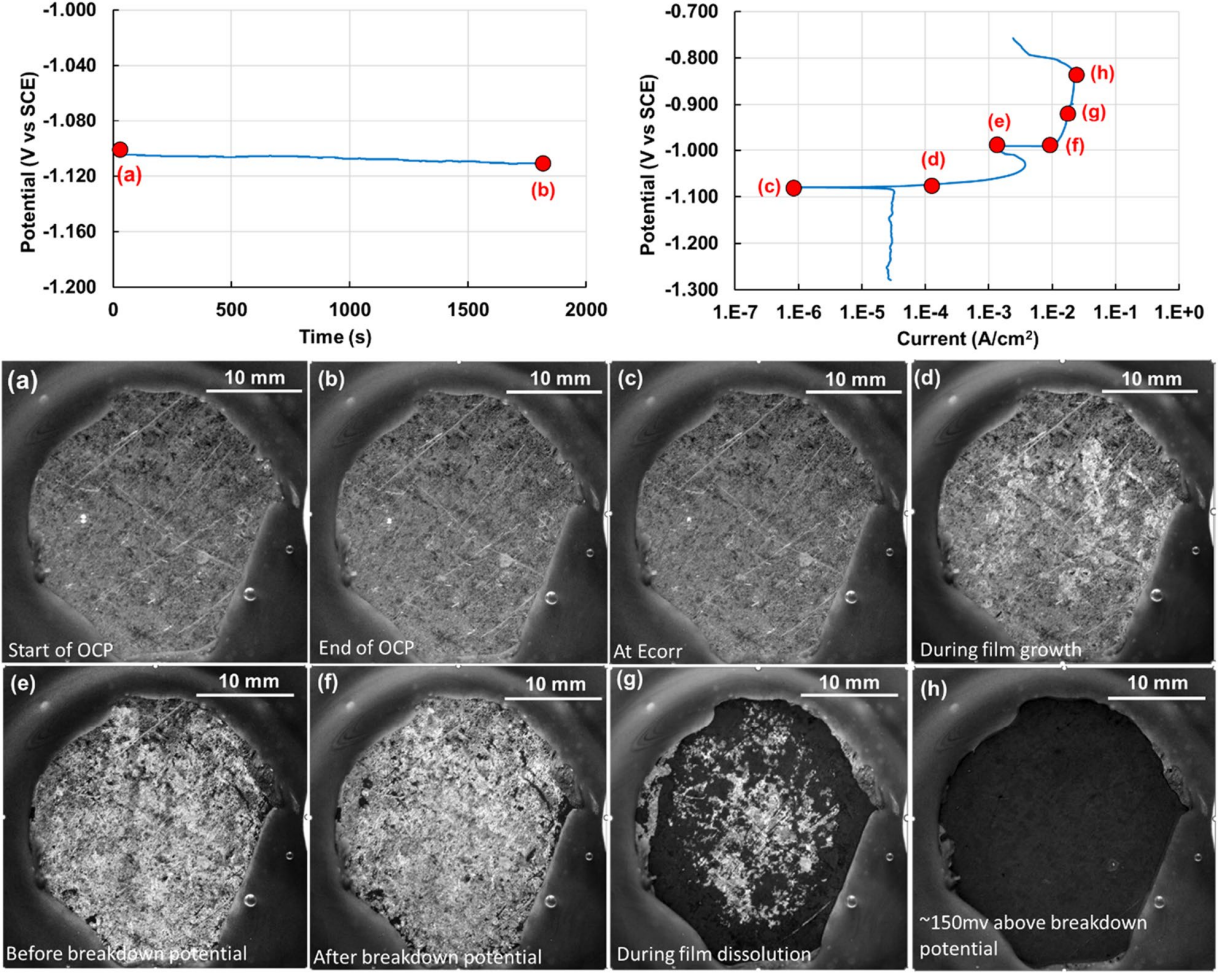
Representative OCP (Potential (V vs SCE) vs time(s)) and PD curves (potential (V vs SCE) vs current(A/cm^2^)) of a galvanized DP590 steel. Annotations highlight locations where images of the sample surface are shown, i.e. (**a**) at the start of OCP, (**b**) at the end of OCP, (**c**) at E_corr_, (**d**) during hydroxide film growth, (**e**) after film growth is completed or just before breakdown of the film, (**f**) after film breakdown, (**g**) during film dissolution, (**h**) ~ 150 mV above the breakdown potential. Images show the formation and dissolution of the light colored Zn(OH)_2_ coating on the surface.

Figure 6 shows representative OCP and PD curves for the galvanized DP590 sample. Annotations in the OCP and PD curves highlight locations where snippet images from the sample’s corrosion video are shown. No resolvable change was observed on the surface of the sample during the OCP curve and during the cathodic polarization curve, as shown in Fig. 6a–c. On increasing the potential into the anodic polarization curve, a surface layer of Zn(OH)_2_ (light gray pixels—Fig. 6d) covered the entire surface of the sample until point (e) on the PD curve (Fig. 6e), i.e. immediately before the breakdown potential. Following this, the hydroxide layer was observed to dissolve as seen in Fig. 6f–h to leave behind the pristine DP590 steel. A video correlating the OCP and PD curves with the video of the corroding sample is included in the supplementary section (DP590_MmC corrosion-best contrast.mp4).

In this case, the combination of imaging and electrochemical measurement is significant for two reasons. First, it provides direct visual confirmation of the formation of a protective hydroxide layer that covers the entirety of the surface just as the corrosion current begins to stagnate and decrease (immediately before point (e) on the curve), indicative of surface passivation. Second, it provides a direct visual confirmation of the presence and the effect of a breakdown potential (at point (e) on the PD curve) where a sharp increase in corrosion current is observed for a small increase in applied potential. Simultaneously, the dissolution of the hydroxide film is observed to start from the areas of the sample near the lacquer until the pristine DP590 steel is left behind (dark pixels in figure (g) and point (h) on the PD curve). The half reactions involved in the corrosion of zinc are as follows:

$${\text{Anodic reaction}}:{\text{Zn}} \to {\text{Zn}}^{{{2} + }} + {\text{ 2e}}^{ - } ,$$

$$\begin{aligned} {\text{Cathodic reactions}}: \, & {\text{HER}}:{\text{ 2H}}_{{2}} {\text{O }} + {\text{ 2e}}^{ - } \to {\text{2OH}}^{ - } + {\text{ H}}_{{2}} \uparrow \\& {\text{ORR}}:{\text{O}}_{{2}} + {\text{ 4e}}^{ - } + {\text{ 2H}}_{{2}} {\text{O}} \to {\text{4 OH}}^{ - } , \\ \end{aligned}$$

$${\text{Net redox reaction}}:{\text{ Zn }} + {\text{ 2H}}_{{2}} {\text{O}} \to {\text{Zn }}\left( {{\text{OH}}} \right)_{{2}} + {\text{ H}}_{{2}} \uparrow .$$

### Case IV: Commercially pure zinc

**Figure 7:**
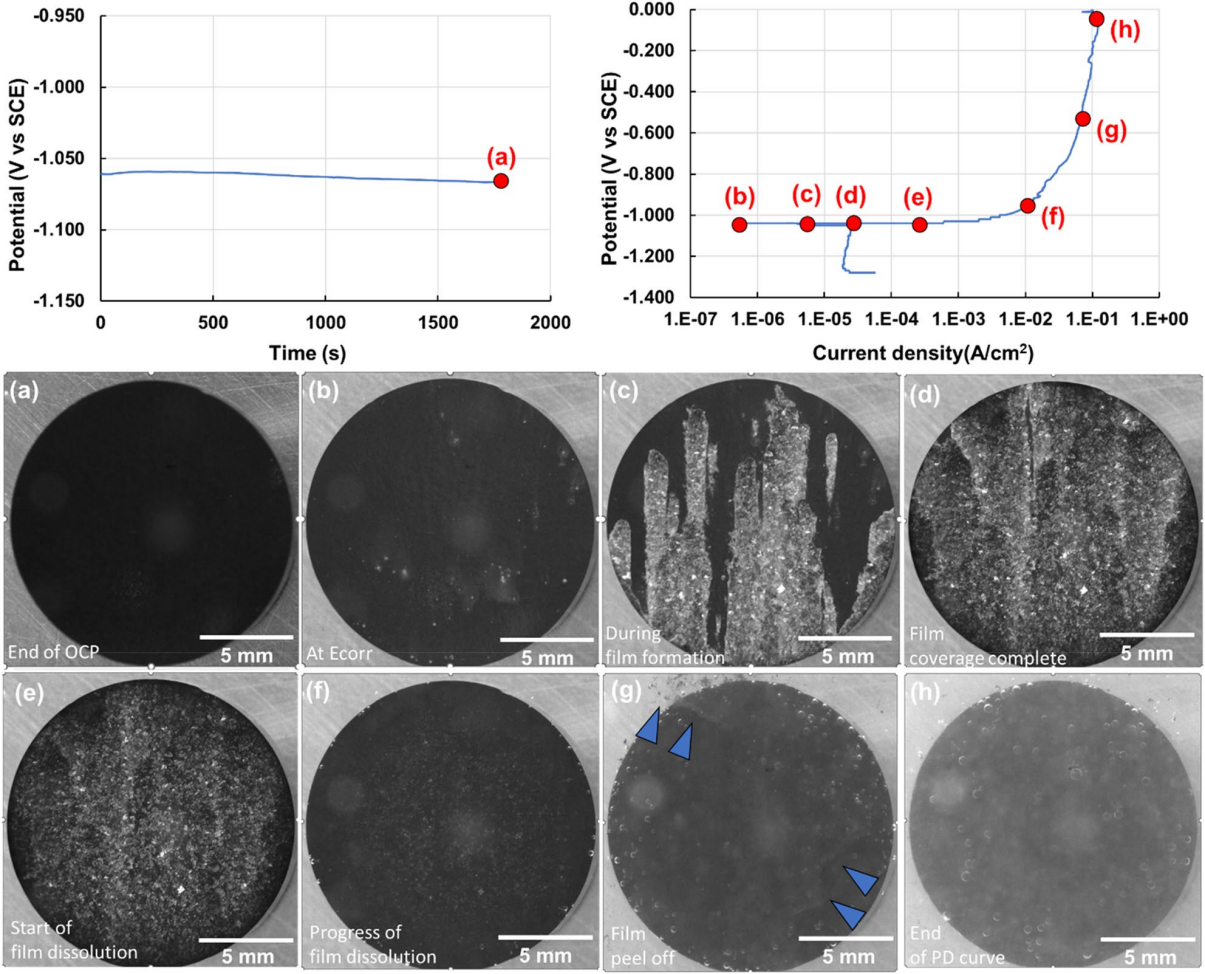
Representative OCP (Potential (V vs SCE) vs time(s)) and PD curves (potential (V vs SCE) vs current(A/cm^2^)) of a pure Zinc sample. Annotations highlight locations where images of the sample surface are shown, i.e. (**a**) at the end of OCP, (**b**) at E_corr_, (**c**) at an applied overpotential of 10 mV above OCP, immediately after Zn(OH)_2_ begins to cover the surface, (**d**) at an applied overpotential of 20 mV above OCP during the completion of coverage of surface hydroxide, (**e**) at an applied overpotential of 30 mV above OCP when the hydroxide layer has started to dissolve, (**f**) at an applied overpotential of 50 mV above OCP when film dissolution appears to be nearly complete, (**g**) at an applied overpotential of 500 mV above OCP during peel off of thin remnant surface film, (**h**) at the end of the recorded PD curve accompanied by surface dissolution and hydrogen release. Images show the increase in corrosion attack as well as hydrogen bubble evolution as a function of imposed potential.

Circular samples of commercially pure zinc were obtained from a circular cross section rod with a diameter of 15.875 mm. The samples were mounted in epoxy and polished to a 1 µm diamond finish. Figure 7 shows the representative OCP, and PD curves obtained from one such sample in 0.6 wt% NaCl solution. The OCP measurements stabilized at a potential of − 1070 mV after 1800 s, following which the PD measurement was performed. During the OCP measurement, no change was observed on the sample surface as seen in inset image (a). Cathodic polarization led to very minor surface color changes that can be seen in inset image (b). The possible set of corrosion reactions are shown below. Included is the oxygen reduction reaction owing to the presence of dissolved oxygen in the electrolyte. It is not possible to determine the dominant cathodic reaction from using the multimodal system in its current configuration.

$${\text{Anodic Reaction}}:{\text{Zn}} \to {\text{Zn}}^{{{2} + }} + {\text{ 2e}}^{ - } ,$$

$$\begin{aligned} {\text{Cathodic reactions}}: \, & {\text{HER}}:{\text{ 2H}}_{{2}} {\text{O }} + {\text{ 2e}}^{ - } \to {\text{2OH}}^{ - } + {\text{ H}}_{{2}} \uparrow \\& {\text{ORR}}:{\text{O}}_{{2}} + {\text{ 4e}}^{ - } + {\text{ 2H}}_{{2}} {\text{O}} \to {\text{4 OH}}^{ - } , \\ \end{aligned}$$

$${\text{Net redox reaction}}:{\text{ Zn }} + {\text{ 2H}}_{{2}} {\text{O}} \to {\text{Zn }}\left( {{\text{OH}}} \right)_{{2}} + {\text{ H}}_{{2}} \uparrow .$$

On increasing the potential beyond the E_corr_, the surface underwent rapid hydroxide formation to form a light gray film of Zn(OH)_2_ that covered the entire surface of the sample. This formation can be seen in Fig. 7c and d. Following further increase in potential, the hydroxide layer is seen to undergo dissolution (Fig. 7e and f) and exfoliate from the sample’s surface as shown by blue arrows in Fig. 7g exposing fresh substrate underneath. This was also accompanied by hydrogen bubble release (Fig. 7g and h), albeit a lower volume of hydrogen compared to that collected in magnesium or aluminum alloys for the range of potentials studied here. A total of less than 0.5 ml of hydrogen was collected during the entirety of this experiment. A video correlating the OCP and PD curves with the video of the corroding sample is included in the supplementary section (Zn-MmC corrosion.mp4).

## Discussion

Analyzing and interpreting results from potentiostat assisted corrosion experiments can be challenging due to the lack of real time spatial and temporal microstructural information from the ongoing corrosion process. The above examples of corrosion from multiple materials systems have shown the beneficial effect of combining the potential assisted corrosion with imaging and hydrogen collection. Imaging the sample has provided insight into the ongoing corrosion processes during OCP and cathodic and anodic polarization. Local differences in hydrogen bubble release can be visualized and used as qualitative analogues for corrosion rate. The total volume of hydrogen collected can also be used to compare the corrosion performance of two or more alloys exposed to the same corrosion condition. In all the materials systems studied here, the application of an anodic overpotential showed the most dynamic changes to the microstructure during corrosion. Processes such as protective film formation during passivation, film dissolution upon increase in potential (as seen in DP590 steel and pure Zn), initiation and propagation of pitting and filiform corrosion under accelerated and open circuit conditions (as seen in AZ91), and localized hydrogen bubble release as an analogue to the local magnitude of corrosion (as in AZ91 and AA6061) have been demonstrated in this study. These observations indicate that ongoing corrosion processes are best observed using multiple synergistic techniques that correlate one another; e.g. the formation and dissolution of a protective film on the surface was observable purely due to imaging as it did not lead to any noticeable changes in the Potentiodynamic polarization curves. Apart from corrosion potential and current, net hydrogen released over a fixed corrosion duration or applied potential range can also be used to rapidly compare the corrosion performance of materials. The relationship between anodic polarization, metal oxidation and hydrogen release are of particular interest in Mg and Al alloys owing to the negative difference effect^2,22,24,30,31^. Additionally, crevice corrosion can also be tracked with the help of the system as the combination of visual observation and hydrogen evolution (trapped or evolving bubbles) can be visual indicators of ongoing crevice corrosion as demonstrated similarly in a recent study^27^. The materials studied here are also widely used for various applications, most noteworthy being in the automotive sector where they can be exposed to harsh corrosive environments (road salt, moisture, galvanic coupling, and varying temperatures). Thus, understanding the precise microstructural changes taking place during corrosion is of vital importance to their performance.

Choosing areas for subsequent analysis after a sample has been subjected to corrosion can be expensive and time consuming due to the lack of adequate information about the microstructural constituents and their behavior during the corrosion process. However, the use of the multimodal corrosion system aids this correlative microscopy workflow for corrosion analysis. By obtaining precise knowledge about the microstructure (e.g. grain size and distribution, phase distribution, intermetallic particle locations, and distribution) prior to corrosion using techniques such as electron backscattered diffraction (EBSD), energy-dispersive spectroscopy (EDS), X-ray microscopy, and scanning electrochemical cell microscopy (SECCM), their precise effect on corrosion initiation and progress can be tracked in real time during the corrosion process^7,32^. The multimodal corrosion system used in this study can highlight the role of local variation in electrochemical properties (relatively cathodic and anodic phases), regions with greater hydrogen release and initiation sites, thus guiding subsequent correlative analysis. Thus, this approach aids in delineating the role of different microstructural features on the material’s overall corrosion behavior.

## Summary

In this study, a novel, simple and versatile (in terms of allowable sample size, shape and whether epoxy mounted or not) corrosion system that combines sample imaging and hydrogen collection during potentiostat assisted corrosion measurements is shown to enable microstructural visualization and analysis *during* the corrosion process. Using examples from four alloy systems (Galvanized DP590 steel, aluminum alloy AA6061, Mg alloy AZ91, and pure Zn), we have shown that aspects such as locations of corrosion damage initiation, sequence of corrosion initiation and propagation, and a qualitative and quantitative understanding of rate of corrosion taking place locally can be obtained. Key events in the corrosion process such as surface film formation during passivation, film breakdown, and film exfoliation have also been demonstrated with direct visual confirmation. Finally, the observation of hydrogen bubble release and its subsequent capture serve as visual indicators of locations of corrosion progress (or the lack of) and total corrosion damage in the microstructure. The multimodal approach also informs of critical locations of the microstructure that can be further analyzed using correlative 2-D or 3-D microscopy. This simple and unique tool can aid industry and academia to understand the overall electrochemical behavior of materials.

### Supplementary Information

Supplementary Video 1.

Supplementary Video 2.

Supplementary Video 3.

Supplementary Video 4.

Supplementary Information 1.

Supplementary Information 2.

Supplementary Figure S1.

Supplementary Video 5.

## Data Availability

The raw OCP and PD data that was acquired during each corrosion experiment has been attached in the supplementary section.
