# Supplementary material for: Probing corrosion using a simple and versatile in situ multimodal corrosion measurement system
Source: Sci Rep. 2023 Oct 4;13:16695. doi: 10.1038/s41598-023-42249-0 (PMC10550931; doi:10.1038/s41598-023-42249-0)
Supplement: Supplementary file 5 — Supplementary Information 1. [file 41598_2023_42249_MOESM5_ESM.docx]

**Supplementary Information**

- Videos showing corrosion of the material systems are included.
- Supplementary Figure S1: Schematic showing the Top and Side views of the bath and associated components.
- Supplementary Data: Excel file containing the raw OCP and PD data from each sample in separate sheets.
