# Supplementary figures and images for: Probing corrosion using a simple and versatile in situ multimodal corrosion measurement system
Source: Sci Rep. 2023 Oct 4;13:16695. doi: 10.1038/s41598-023-42249-0 (PMC10550931; doi:10.1038/s41598-023-42249-0)

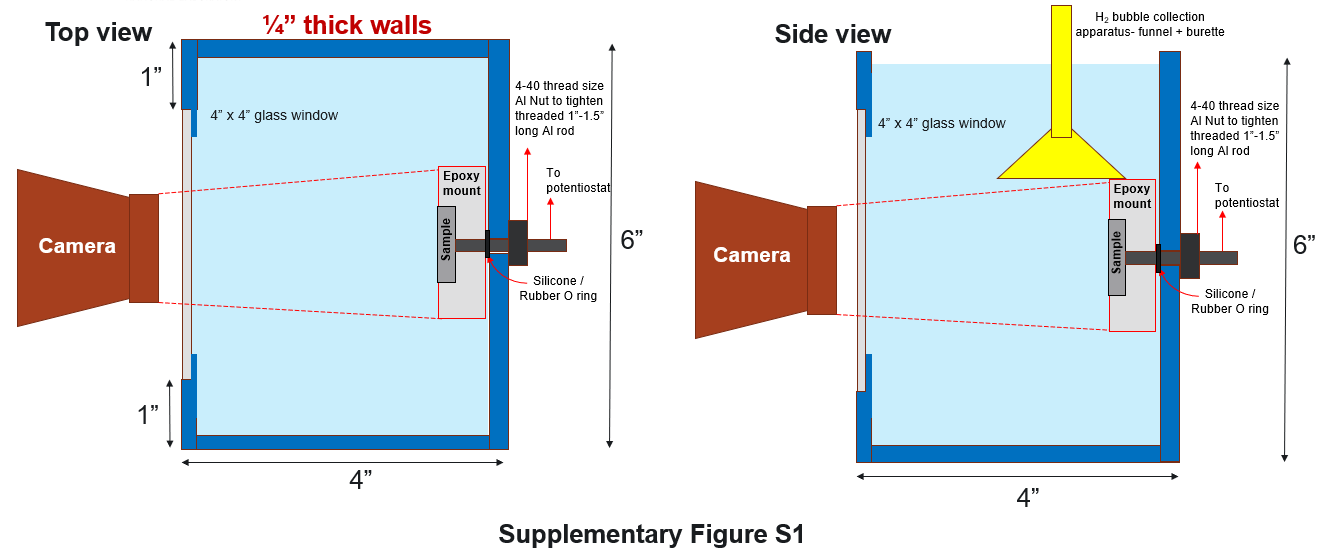

Supplement: Supplementary file 7 — Supplementary Figure S1. [file 41598_2023_42249_MOESM7_ESM.png]
